# A Novel Behavioral Fish Model of Nociception for Testing Analgesics

**DOI:** 10.3390/ph4040665

**Published:** 2011-04-18

**Authors:** Ana D. Correia, Sérgio R. Cunha, Martin Scholze, E. Don Stevens

**Affiliations:** 1 Instituto de Medicina Molecular (IMM), Faculdade de Medicina, Universidade de Lisboa, Lisboa, 1649-028, Portugal; 2 Faculdade de Engenharia, Universidade do Porto, Porto, 4200-465, Portugal; 3 Centre for Toxicology, The School of Pharmacy, University of London, London, WC1N 1AX, UK; 4 Department of Biomedical Science, UPEI-AVC, Charlottetown, PE, C1A4P3, Canada

**Keywords:** morphine, fish, dose-response, zebrafish

## Abstract

Pain is a major symptom in many medical conditions, and often interferes significantly with a person's quality of life. Although a priority topic in medical research for many years, there are still few analgesic drugs approved for clinical use. One reason is the lack of appropriate animal models that faithfully represent relevant hallmarks associated with human pain. Here we propose zebrafish (*Danio rerio*) as a novel short-term behavioral model of nociception, and analyse its sensitivity and robustness. Firstly, we injected two different doses of acetic acid as the noxious stimulus. We studied individual locomotor responses of fish to a threshold level of nociception using two recording systems: a video tracking system and an electric biosensor (the MOBS system). We showed that an injection dose of 10% acetic acid resulted in a change in behavior that could be used to study nociception. Secondly, we validated our behavioral model by investigating the effect of the analgesic morphine. In time-course studies, first we looked at the dose-response relationship of morphine and then tested whether the effect of morphine could be modulated by naloxone, an opioid antagonist. Our results suggest that a change in behavioral responses of zebrafish to acetic acid is a reasonable model to test analgesics. The response scales with stimulus intensity, is attenuated by morphine, and the analgesic effect of morphine is blocked with naloxone. The change in behavior of zebrafish associated with the noxious stimulus can be monitored with an electric biosensor that measures changes in water impedance.

## 1. Introduction

Pain research and its translation into improved diagnosis and therapy both have a high priority because of the increasing incidence of pain disorders with ageing [1,2]. In spite of this, progress in neuropharmacology and therapeutics worldwide is largely stalled, with only a small number of substances qualifying for clinical use [3]. This is in part due to the lack of appropriate animal models to mimic these conditions and to assess the validity of candidate drugs.

Mammalian models are commonly used to develop safer and more effective pain treatments for patients. However, this is a controversial issue from an ethical point of view and has encouraged the development of alternative non-mammalian models. There are reports using fish, amphibians, reptiles or birds to understand the mechanisms of opioid analgesia and also to test the selectivity of analgesic drugs [4,5]. Fish possess the necessary sensory components to detect a noxious stimulus and also have brain areas similar to those of higher vertebrates that respond to painful stimuli [6], although some argue that the responses are simply reflexive [7]. The neuronal activity detected in fish brain centers such as the spine, cerebellum, tectum and telencephalon also suggests the possibility of pain perception in fish in which the dorsal telencephalon (similar to the cortex in mammals) may mediate the coordination of the pain information [8,9]. As a result of these observations, teleost fish have been used as models for pain and analgesia research [10,12-15]. However, only few fish species have been used to test the efficacy of opioids and non-opioids [12,14-16].

We selected zebrafish (*Danio rerio*) as a test species because its nervous system shares many fundamental similarities with other vertebrates, including the opioid system [17,18]. We used zebrafish to develop an acute behavioral nociceptive test that monitors individual swimming responses to a noxious chemical stimulus. On the basis of an electronic biosensor device (MOBS), locomotor responses to acetic acid were recorded, a noxious agent that reduces activity of zebrafish [13]. These nociceptive responses to acetic acid are the basis of our model and we investigated in time-course studies its applicability and robustness with treatments of an analgesic drug (morphine) and an opioid receptor competitive antagonist (naloxone). First we tested two different doses of acetic acid to estimate the optimal dose. As “optimal” we defined a dose that reduced fish activity in a stable manner long enough to do an experiment, but was not an overdose resulting in freezing behavior or death. A strong difference between control and nociceptive fish responses is also important from a statistical point of view, as it supports the detection of significant changes to nociceptive activity (e.g., in response to further drug treatments), especially as behavioral endpoints are typically exhibit high variance. Our dose-finding study was performed with two recording systems, an electric biosensor (the MOBS system) and by video tracking. The latter was used to confirm the results of the automatic system, which then was used as standard recording system in subsequent studies.

Next, we investigated whether our system was able to identify anti-nociceptive responses caused by two doses of morphine. Although the primary aim of our study was to detect changes from the nociceptive response baseline, we also were interested whether dose-response pattern could be detected. In the last study we tested whether the effect of morphine could be attenuated by naloxone, a drug that is commonly used to counter the effects of opiate overdoses. In our study, zebrafish were exposed to three different agents, an experimental situation that we considered very demanding of our test system.

## 2. Material and Methods

### 2.1. Chemicals

All chemicals were purchased from Sigma-Aldrich (St. Louis, MO, USA). Solutions of acetic acid (5 and 10%) were dissolved in deionised water and morphine and naloxone solutions in sterile saline (0.9% NaCl). Buffered tricaine methanesulfonate (MS222) was used to anesthetize the fish.

### 2.2. Test Animals

The zebrafish (*D. rerio* Hamilton 1822) strain used for this work was the AB line (Zebrafish Facility, IMM, Portugal). Animals were maintained under standard conditions and experiments were approved by the Institutional Animal Care and Use Committee.

### 2.3. Behavioral Assay

#### 2.3.1. General Protocol

Before the experiments, small groups of male fish (10-15 animals, body weight 0.5 ± 1g) were acclimatized to the experimental testing conditions (temperature 22 °C ± 1 °C, 10 h:12 h light-dark cycle) in 17 liter glass aquaria under static conditions and for a minimum of one week. Food was not provided 24 h before or during the experiments. On the day of experiments, either the treated or control groups of fish were placed individually in the test chambers supplied with oxygenated tap water (22 °C ± 1 °C). Ungrouped fish were acclimated to the test chambers for 30 min and then individual baseline responses (pre-treatment) were monitored for one hour between 10 and 12 a.m. Each fish was individually anesthetized using tricaine (50 mg/L) and then was injected with the test substance(s). During the injection they were in a medium-to deep-plane level of anaesthesia and had lost their reflex responses and muscular control. Afterwards they returned to their original test chambers and allowed 30 min to recover from the anaesthesia. Individual fish swimming responses (post-treatment) were then recorded for 80 min, *i.e.*, 110 min after the injection. We recorded individual fish activity from four fish per day, as we considered this sample size as best to minimize the delays due to the injection scheme in relation to the simultaneous recording of all sample fish in the MOBS system. After behavioral recording, fish were sacrificed humanely by concussion followed by severing the spinal cord. The behavioral experiments always were performed by the same experimenter.

#### 2.3.2. MOBS System

A detailed description of this equipment is reported elsewhere [11]. Briefly, the system consists of a hardware device, a set of four experimental test chambers (cylindrical chambers, 6 cm in diameter, 10 cm long) and signal processing software running on an external computer. The device injects weak analog electrical signals into the water of the test chambers through a pair of non-invasive stainless steel electrodes. The shape and spectrum of these signals are generated by the software in the digital domain. The response is measured as a change in impedance of the water column received by another pair of non-invasive stainless steel electrodes associated with movements of the fish. These recorded signals are amplified and converted back into the digital domain at 48 kHz, filtered, demodulated and down-sampled to 100 Hz. Upon processing, the system provides a signal in the frequency band of 0.2 Hz to 40 Hz that is correlated with the fish activity. In preliminary studies we adjusted the software of the MOBS system to a specific locomotion behavior of zebrafish, with series of bursts in the domain of MOBS corresponding to the tail-flip activity of zebrafish. Thus the outcomes from MOBS reflect the number of tail-flips per minute per individual fish.

#### 2.3.3. Video Recording System

Individual fish were placed in a small tank (9 cm × 8 cm) and their swimming behavior recorded with a video camera positioned over the tank. Online records were acquired in movies of 1 min length (600 frames), saved on the computer and later analyzed for speed activity (mm/sec) using the software Image Pro-Plus^®^. Speed was calculated as the change in position in two dimensions.

During the experiments, the pre-treatment behavior of individual fish was recorded for 15 min before anesthesia. After a recovery period of 30 min, post-treatment behavior was recording every 10 min (10 movies of 600 frames each) at intervals of 30 min after recovery (three sections of records were done). The experimenter was not visible by the fish during recording.

### 2.4. Experimental Studies

#### 2.4.1. Study 1—Zebrafish Behavioral Model of Nociception

The purpose of this study was to identify an effective dose of acetic acid without losing fish due to freezing behavior or mortality. Two doses of acetic acid (5% and 10%) were selected on the basis of literature data [13] and tested using the MOBS system. The higher dose was favored as default nociceptive dose for the final model under the prerequisite of: (1) significantly lower fish activity compared with the responses to 5% acetic acid, (2) stable response over the recording period, and (3) no higher loss of fish than for the controls.

**Figure 1:**
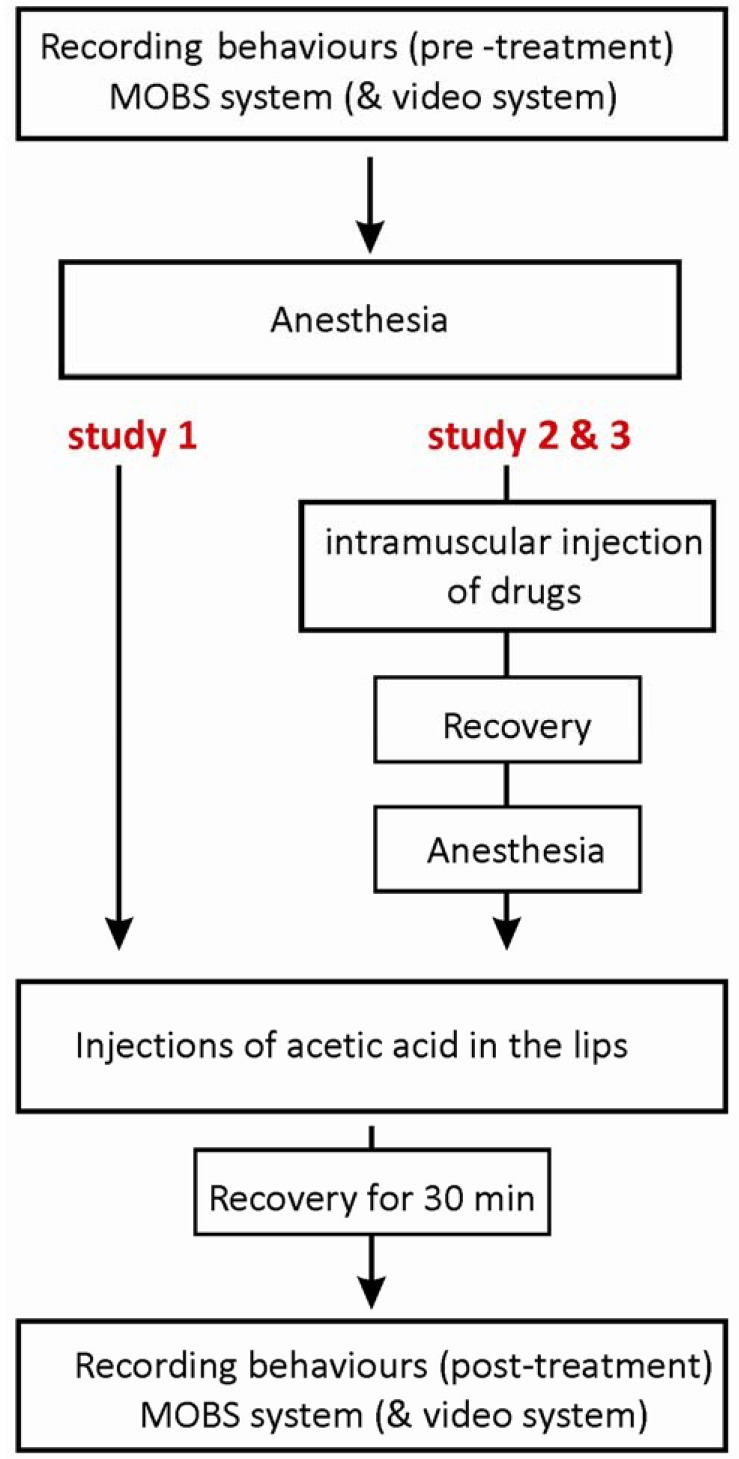
Experimental study design.

The acetic acid was carefully injected with a total volume of 5 μL per 0.5 g fish (5%, n = 6 or 10%, n = 8) into the top and bottom frontal lips of fish using a gastight syringe and a 30-gauge needle (Hamilton, Reno, NV, USA). The control group (n = 14) was injected with a sterile saline solution. The study with the MOBS system consisted of six experiments, each including two controls and both acetic acid treatments, and one experiment with two controls and two 10% acetic acid treatments. For the video tracking system, the injection procedure was the same, and again we tested four fish per day, with 8 experimental runs with two controls and two 10% acetic acid treatments, and a final run with saline only treatments. The protocol of the injection procedure is shown in Figure 1.

#### 2.4.2. Study 2—Effect of Morphine on Zebrafish Nociceptive Behavior

The aim of study 2 was to test the efficacy of morphine to induce anti-nociceptive behavior in the zebrafish model. Fish were first treated with two morphine doses (3 and 6 mg/kg) and afterwards stimulated with acetic acid using the same procedure as the first study. The two morphine doses were selected on the bases of literature data [19]. In addition, we also tested a control group of fish, treated with a saline solution instead of morphine. All three treatments were administered intramuscularly into anesthetized fish, with morphine in a total volume of 3.0 μL per 0.5 g fish by RN gastight syringe (Hamilton). Two control treatment groups were tested, the first with morphine only, and the second with saline only, *i.e.*, fish from both these control groups were not exposed to acetic acid. The dosing schemes for this as well other all studies are shown in Table 1.

#### 2.4.3. Study 3—Modulation of the Analgesic Effect of Morphine with Naloxone

The purpose of study 3 was to investigate whether anti-nociceptive behavior induced by morphine could be altered by naloxone, an antagonist of mu-opioid receptors. Two treatment groups were tested, with fish receiving either 6 mg/kg morphine together with saline solution or 6 mg/kg morphine together with 6 mg/kg naloxone. Morphine and naloxone (or saline) were co-injected intramuscularly (6 mg/kg: 6 mg/kg, in a total volume of 6 μL) in pre-anesthetized fish by using a RN gastight syringe (Hamilton). Fish from both groups were later treated with 10% acetic acid injections to the lips. The protocol of the injection procedure is illustrated in Figure 1, and the dosing scheme together with numbers of fish tested is shown in Table 1.

### 2.5. Data Analysis

**Figure 2:**
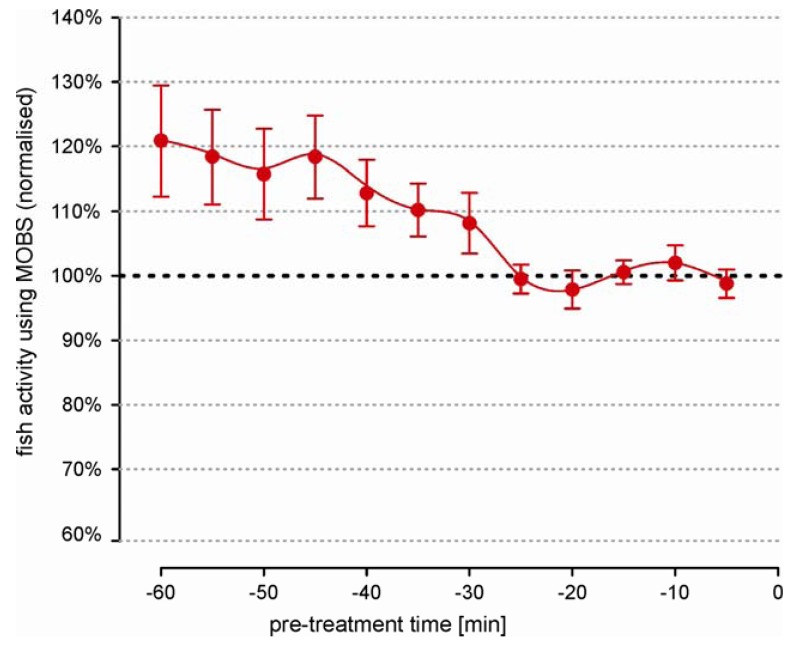
Pre-treatment activity of zebrafish recorded by the MOBS system. Data have been normalized to the median of readings from the last 25 min and are shown as Winsorized mean ± standard error (N = 27). Values when activity had stabilized were used to normalize the data for each fish.

Behavioral data were normalized on a fish-by-fish basis to the medians of their individual pre-treatment values and expressed as a percentage, so all results describe changes relative to the median of pre-treatment values. Pre-treatment values should be a representative description of the individual fish activity, but fish tended to be more active at the beginning of the 30 min recording period. Thus, for each fish we used the pretreatment values when they became stable (see Figure 2). All data from the same study and treatment from different days were pooled for further statistical data analysis. For graphical presentation, data were expressed as the 20% Winsorized mean with its standard error [20].This estimator is less sensitive to outliers as it uses the 10% highest and 10% lowest value to replace the most extreme values. The effect of treatments on the changes of zebrafish behaviors across the recording sessions was analyzed using a nonparametric mixed-effect model for longitudinal data [21]. This model is more robust against extreme values that occur when recording behavioral activity. The level of statistical significance was set to p < 0.05. All statistical analysis was performed using the SAS statistical software (SAS Institute Inc., Cary, NC, USA).

## 3. Results

Due to the experimental setup only four fish per day were tested and data from different test days were used. As a consequence we cannot exclude the possibility that the variation in our data might have increased (inter-day variation), but we found no indications of a systematic trend. The fish numbers per treatment are shown in Table 1.

### 3.1. Pre-Treatment Behavior

We observed large differences in activity level between untreated individual fish that were independent of the length of the recording time. This prevented the data pooling of the absolute measurements. Therefore we used relative changes (ratio) between the individual post-treatment values and the pre-treatment activity. Statistically, a ratio is very sensitive to small errors in the estimation of the pre-treatment median, which meant that the individual fish behavior must be representative and stable. Figure 2 summarizes the pre-treatment activity from 26 fish over a 60 minute period. Individual measurements from MOBS were normalized to the median of values from the last 20 min because the average fish activity was most stable in this time period.

Earlier a clear negative trend is visible, with fish reducing their activity by an average of about 20%. Also the error bars are 6 times larger at beginning of the recording than in the last 20 min. These observations strongly imply that the recording of pre-treatment behavior should be performed over a sufficiently long time period in order to avoid a biased estimation of treatment responses.

### 3.2. Zebrafish Behavioral Model of Nociception

**Figure 3:**
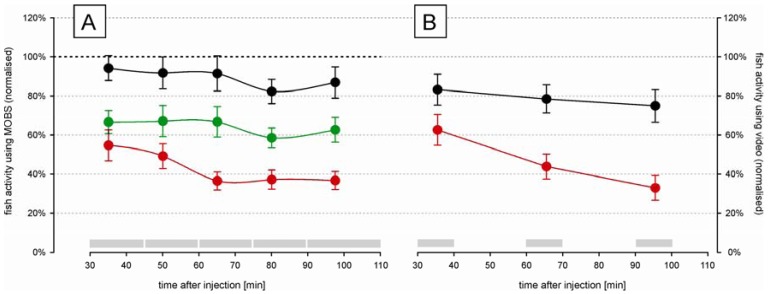
Time-course changes in zebrafish activity after injection of 10% acetic acid (red), 5% acetic acid (green) and control saline solution (black). Fish activity was recorded using the MOBS system (A) and by video analysis (B). All values are normalized to the median of pre-treatment activity (100%). Fish activities for periods of 15 min and 10 min (MOBS system (A) and video recording (B), respectively) are shown as Winsorized mean ± standard error, connected with a solid line. Recorded time periods are highlighted as grey bars above the time scale.

The time-course changes in zebrafish activity are shown in Figure 3A after injection of two doses of acetic acid or saline into the lips. They were recorded by the MOBS system for 80 min after a recovery period of 30 min after injection. Both treatments resulted in a significant decrease in activity (p < 0.01), and the three treatments (saline control, 5%, 10%) all differed significantly from one another (p < 0.01). Saline control fish were less active than before treatment (black symbols, Figure 3A) but this decrease was not statistically significant. Fish treated with 10% acetic acid showed a robust and stable decrease in fish activity for 60 min after injection. Therefore, we used 10% acetic acid injection as the noxious stimulus in all subsequent studies.

In separate trials we measured fish activity using a video camera (Figure 3B). Similar to that recorded with the MOBS system, the 10% acid injection resulted in a significant decrease in fish activity. In these trials activity continued to decrease throughout the observation period and the slopes of the decrease in activity with time differed between saline control and 10% acetic acid injection (p < 0.001). The saline controls recovered only to 80% from their pre-treatment activity, whereas the acid treated fish showed at the end decreased activities that were comparable to those recorded by MOBS.

### 3.3. Effect of Morphine on Zebrafish Nociceptive Behavior

**Figure 4:**
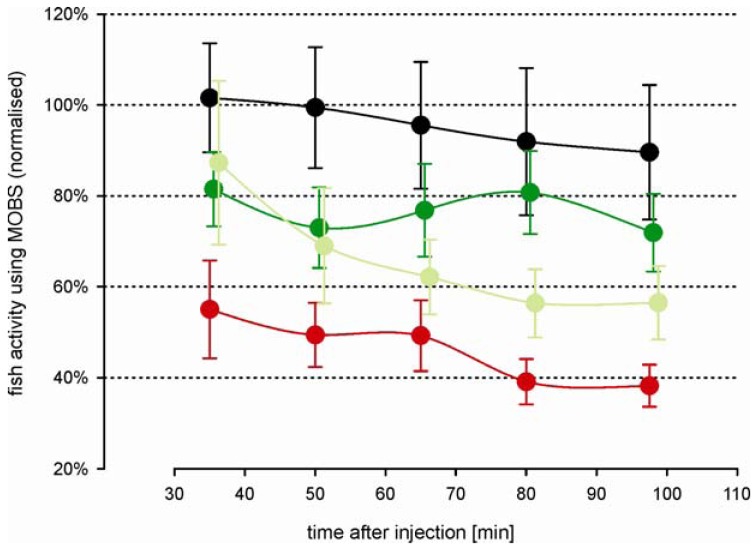
Time-course changes in zebrafish activity associated with a noxious stimulus (10% acetic acid lip injection) to intramuscular treatments with 0 mg/kg morphine (red), 3 mg/kg morphine (light green) and 6 mg/kg morphine (dark green). In addition, a group of control fish with no noxious stimulus was treated with saline solution (black). All values were recorded using the MOBS system and are normalized to the median of pre-treatment activity (100%). Fish activities for periods of 15 min are shown as Winsorized mean ± standard error, connected by a solid line.

**Figure 5:**
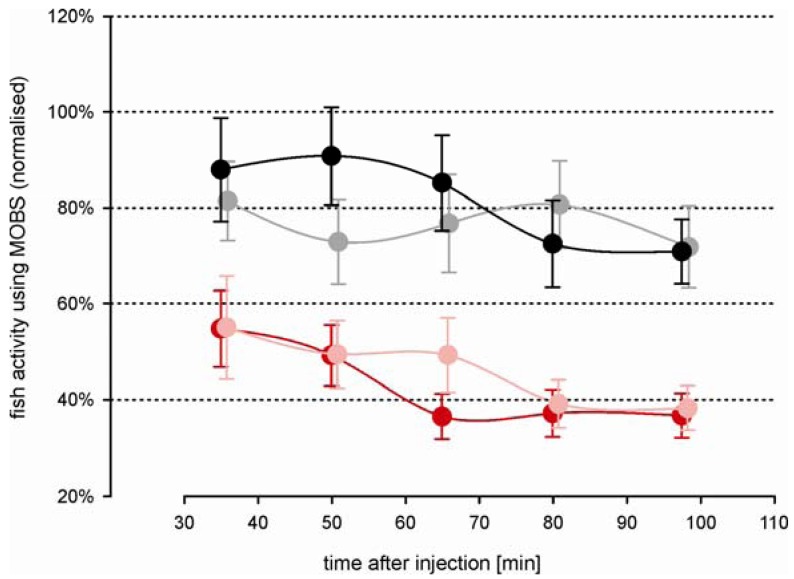
Impact of intramuscular saline solution on the time-course changes in zebrafish activity. Fish were treated with 10% acetic acid injection alone (dark red), in combination with 3μL saline solution (intramuscular injection, light red), in combination with 6 mg/kg morphine (intramuscular injection, gray), and in combination with 6 mg/kg morphine and 3μL saline solution (black). Treatments are from different studies, all values were recorded using the MOBS system and are normalized to the median of pre-treatment activity (100%). Fish activities for periods of 15 min are shown as Winsorized mean ± standard error, connected with a solid line.

Morphine injected prior to the acid injection attenuated the decrease in activity associated with the noxious stimulus (Figure 4). Both morphine treatments resulted in a significant increased activity compared with the acid treated fish, but not differ significantly from one another (p = 0.12). However, this is largely because there was no difference between doses during the first 60 min of the recording period. The difference between doses was significant considering only the last 60 min of the recording period. Saline control fish responded with nearly the same activity as measured before treatment. Compared with the outcomes from the first study, the variation in all treatment groups was greater, possibly reflecting a higher degree of individual behavior pattern to the more complex injection procedure. However, the fish responses to the noxious acid stimulus were in good agreement between both studies (Figure 5); there were no statistical significant differences between them. Our results show that the model has good reproducibility and is robust. Morphine alone (*i.e.*, in the absence of any noxious stimulus) resulted in a small decrease in fish activity but activity levels did not differ from saline controls (data not shown). That is, morphine in and of itself, did not have a significant effect.

### 3.4. Modulation of the Analgesic Morphine Effect through Naloxone

**Figure 6:**
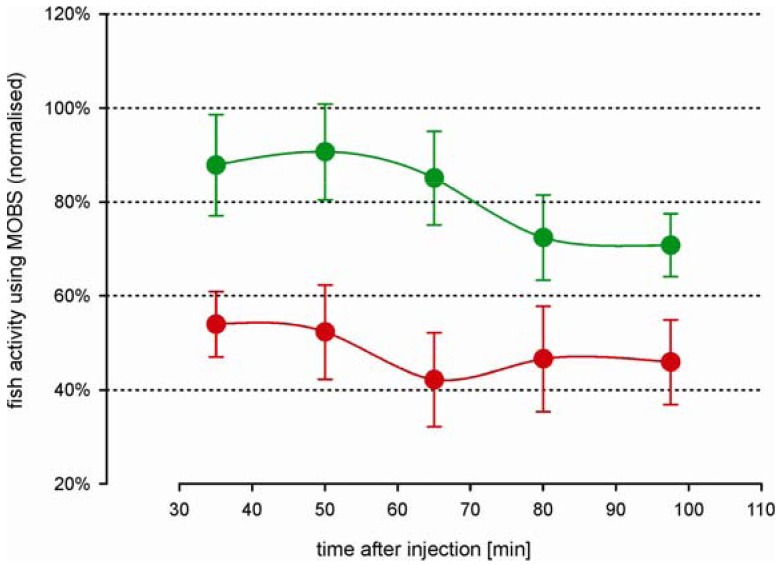
Time-course changes in zebrafish activity to 0 mg/kg naloxone (green) and 6 mg/kg naloxone (red). All fish were treated with a noxious stimulus (10% acetic acid injection) that was attenuated by 6 mg/kg morphine (intramuscular injection). All values were recorded using the MOBS system and are normalized to the median of pre-treatment activity (100%). Fish activities for periods of 15 min are shown as Winsorized mean ± standard error, connected by a solid line.

The decrease in fish activity caused by the injection of 10% acetic acid was attenuated by a prior injection of 6 mg/kg morphine (green symbols, Figure 6), resulting in a stable activity level of about 70% at the end of the recording period. Compared with fish from study 2 that were treated intramuscularly only with morphine, these results were very similar (Figure 5, black and gray symbols), indicating that the saline solution had no significant impact on the outcomes. The attenuation of the decrease in activity was blocked when naloxone was injected at the same time as the morphine (red symbols, Figure 6): the treatments of morphine with and without naloxone were significantly different at all time periods after the acid injection. The average activity from the naloxone and morphine treated fish were only slightly higher than from fish that were treated by only acid lip injection (red symbols, Figure 3A), suggesting that naloxone counteracted the effects of morphine.

## 4. Discussion

Current knowledge about nociception in fish is limited and our present study provides relevant data about the capacity of zebrafish for nociception and the efficacy of morphine as an analgesic and naloxone as antagonistic substance. Our main goal was to test if the locomotor response to a noxious chemical stimulus in zebrafish is a sensitive and robust model that can be used to evaluate analgesics. In particular, does the response scale with the intensity of the stimulus? Does morphine attenuate the response to the noxious stimulus in a dose dependent manner? Is the effect of morphine blocked with naloxone? In addition we were interested in assessing the value of an automated monitoring system (MOBS) for the zebrafish model by comparing it with traditional video techniques.

### 4.1. Zebrafish Behavioral Responses to a Chemical Stimulus

The utility of non-mammalian models in the study of pain has been presented before, especially by Stevens [4]. The particular advantages of using zebrafish as a model in chemical screens have been reviewed by Zon and colleagues who argued that there is no better vertebrate suited to high-throughput phenotyping [22,23]. The main advantages are their cost-effectiveness, large number of offspring (up to 300 per female) and abundance of molecular and genomic tools.

One challenge in pain studies in a novel animal model is the choice of the nociceptive stimulus. The many advantages and disadvantages of chemical, thermal, force or electrical stressors have been reviewed by Le Bars *et al.* [24]. There is convincing evidence that fish possess nociceptors that react in a fashion similar to those in mammals [6,25,26]. Moreover, there is some evidence regarding the pathway from peripheral nociceptors to the brain [8,9]. Recently acetic acid has been used to stimulate peripheral nociceptors in fish [25,27] and in one study in zebrafish. Reilly *et al.* [13] reported that 5% acetic acid resulted in a decrease in activity in zebrafish and our study adds to this showing that the magnitude of the behavioral response scales with the concentration of acetic acid. These findings suggest that zebrafish detected the acetic acid as a nociceptive stimulus and responded to it with a change in behavior. Most studies using acetic acid in mammals inject the noxious stimulus IP and measure writhing. One study compared responses to various chemical noxious stimuli injected into the paw of the rat and showed that responses to acetic acid are similar to those to formalin [28]. In mammals the responses are flinching and licking the injected paw. In rats, these responses are marked during the first 10 min followed by a much less intense response for 20 to 65 min. The decrease in activity that we saw in zebrafish in the present experiment lasted throughout the 110 min recording period and we do not know how long it would have persisted.

### 4.2. The Effect of Morphine in the Zebrafish Model of Nociception

Considering that little is known about analgesics in fish [4], we tested the effect of two doses of morphine in our zebrafish nociceptive-based model. We observed that the administration of morphine sulphate, 30 min prior to giving the acetic acid noxious stimulus, attenuated the nociceptive-related behaviors of zebrafish in a dose-dependent manner. Previous studies using acetic acid as a noxious stimulus have reported the analgesic effect of morphine in fish but did not test the effect of dose [14,15,19] and in one case used an extraordinarily high dose of morphine (3,000 mg/kg, [27]). Morphine at either 40 mg/kg or 50 mg/kg dose did not alter the acute response to a thermal noxious stimulus in goldfish, but seemed to counteract test-induced changes in behavior [29]. The doses used in our study are close to the range (1-10 mg/kg) commonly reported to elicit analgesic effects in mammals [30,31].

### 4.3. Modulation of Antinociceptive Behavior by Naloxone

We also showed that pre-treatment with naloxone, an opioid receptor antagonist, significantly attenuated morphine antinociceptive behaviors in adult zebrafish. These results suggest an opioid receptor involvement in the morphine effect similar to mammals [32] and amphibians [33]. Two other studies using fish have reported that naloxone blocks analgesic effects of opioids [10,34]. In mammals, the analgesic effect of opioids is mediated mainly by mu-opioid receptors (MOR), a G-protein coupled receptor expressed in the CNS [35]. MOR homologues have been identified in several teleosts including in zebrafish and appear to be highly conserved in both the sequence and pharmacological properties [18,36,37,38,39].

### 4.4. Automated Analysis of the Nociceptive Behavioral Test in Zebrafish Model

Another critical issue in establishing a model in nociception is the time required to assess the change in behavior in response to the noxious stimulus in the presence and absence of potential analgesics. Behavioral tests are complex and quantifying the behavioral response is time consuming and labor intensive. Automation in this field is crucial because it allows the experimenter to conduct several tests simultaneously. We used an electric biosensor (MOBS) to detect alterations in the locomotor activity in zebrafish before and after the noxious stimulus with and without analgesia. We hypothesized that this automated system would be less labor intensive compared with other systems (*i.e.,* video tracking-system). The MOBS system has several advantages over a video tracking systems such as: i) no additional light sources are required minimizing the likelihood of interfering with the test organism; ii) the signal processing is controlled by external software and allows a flexible adaptation to changing testing conditions and iii) it is independent of the size of the test organism; juveniles as well as larger adults can be analyzed quantitatively at high speed. We showed that the baseline motor activity patterns of zebrafish can be accurately detected by the MOBS system because this species tends to swim with a series of bursts. Each burst is composed, from the signal detection point of view, of a sequence of strong tail-flips, at a rate of 2-5/s. This swimming pattern can be detected in the signal as a series of sharp edges where each edge associated with a tail flip. The detection of these sharp edges in the signal requires recovering a frequency spectrum of at least 20 Hz. Our software performs some pre-processing to perform automatic gain control and to adjust sharpness detection thresholds. Also, procedures are employed to detect and avoid a few situations of false positives that were identified when analyzing the signal shape. It was clear that both from visual inspection and statistical analysis that this measure strongly reflects the locomotion activity level of each fish. In earlier time-course studies we have produced visual ethograms describing the swimming behaviors of zebrafish and these recorded were compared with the signal output (*i.e.*, shape of the waves over time when viewed on an oscilloscope) produced by MOBS in order to assure a correct identification of zebrafish swimming patterns.

To summarize, we showed that the locomotor response to a noxious chemical stimulus in zebrafish is a sensitive and robust model that can be used to evaluate analgesics - the response scaled with the intensity of the stimulus, it was attenuated by morphine in a dose dependent manner and naloxone blocked the analgesic effect of morphine. Furthermore, we confirmed the validity of this system by performing a similar experiment using a video tracking-system. In both situations the activity of fish decreased throughout the trial period after the noxious acetic acid stimulus. It demonstrated that the behavioral response of zebrafish to a noxious stimulus can be automated by MOBS. In the present experimental design we monitored four fish at the same time, but the present system could monitor up to 14 fish [11].

The MOBS system is capable of measuring other behavioral activity patterns such as fish ventilation [11]. However, ventilation could not be measured in the present experimental design because the chambers were too large relative to the size of the fish. One solution to this technical limitation would be using smaller test chambers, but this would confine the fish.

We developed the model primary for qualitative screening purposes, but with an option for further quantitative dose response analysis (e.g., low dose analysis). The selected fish and dose numbers were too small to provide a more accurate dose-response pattern over time, but in principle we believe that this could be achieved with appropriate automation. The main bar for further proceeding is the time-consuming injection procedure, which was in all studies performed by one experimenter and thus the most limiting factor. A further impediment for routine testing is the current lack of appropriate statistical software that provides a quick and robust analysis of behavioral data. Traditional software packages are based on ANOVA methods with simple model assumptions that are often not fulfilled by behavior data. In our experience, the biggest problem are extreme data values (corresponding to high activity bursts within small time periods) that cannot be smoothed by common data transformation techniques, but require more robust statistical methods. We used a nonparametric approach to analyze the longitudinal data programmed in SAS, but to our knowledge no commercial software package is on the market that provides similar or alternative robust methods. This limits not only the analysis of existing dose-response data, but also the planning of necessary sample sizes for further studies. Preliminary results from simulation studies on our data suggest that at least 20 fish per treatment are required to achieve sufficient statistical power to detect at least 10% response differences between two treatments as statistically significant. This number corresponds to the minimal sample size of rodents used in many toxicological studies for testing chemicals on developmental and reproductive toxicity.

## 5. Conclusions

A change in behavioral responses of zebrafish to acetic acid is a reasonable model to test analgesics. The response scales with stimulus intensity, is attenuated by morphine in a dose-dependent manner, and the analgesic effect of morphine is blocked with naloxone. The change in behavior of zebrafish associated with the noxious stimulus can be monitored with an automated system that measures changes in impedance.

## Figures and Tables

**Table 1:** Dosing schemes used with the automatic recording system (MOBS). * N = number of fish used for data analysis, in brackets number tested.

	**Injection**			
	**Intramuscular**		**Lip**	N*
Study 1	---		5 μL saline solution	12 (14)
	---		5 μL acetic acid 5%	6 (6)
	---		5 μL acetic acid 10%	8 (8)

Study 2	3 μL saline solution	+	5 μL acetic acid 10%	10 (10)
	3 μL morphine 3mg/kg	+	5 μL acetic acid 10%	9 (10)
	3 μL morphine 6mg/kg	+	5 μL acetic acid 10%	8 (10)
	3 μL morphine 3mg/kg	+	5 μL saline solution	7 (8)
	3 μL saline solution	+	5 μL saline solution	8 (8)

Study 3	3 μL morphine 6mg/kg	+	5 μL acetic acid 10%	11 (12)
	+ 3 μL naloxone 6mg/kg			
	3 μL morphine 6mg/kg	+	5 μL acetic acid 10%	9 (10)
	+ 3 μL saline solution			

## References

[1] Breivik H., Cherny N., Collett B., Conno F., Filbet M., Foubert A.J., Cohen R., Dow L. (2009). Cancer-related pain: A pan-European survey of prevalence, treatment, and patient attitudes. Ann. Oncol..

[2] Breivik H., Collett B., Ventafridda V., Cohen R., Gallacher D. (2006). Survey of chronic pain in Europe: prevalence, impact on daily life, and treatment. Eur. J. Pain.

[3] Friend S. (2010). Crowdsourcing drug discovery. Scientist.

[4] Stevens C.W., Conn P.M. (2007). Non-Mammalian Models for the Study of Pain. Source Book of Models for Biomedical Research.

[5] Stevens E. Don. (2008). “Pain” and analgesia in fish: what we know, what we don't know, and what we need to know before using analgesics in fish. Blue sky to deep water: the reality and the promise.

[6] Sneddon L.U. (2004). Evolution of nociception in vertebrates: comparative analysis of lower vertebrates. Brain Res. Rev..

[7] Braithwaite V. (2010). Do Fish Feel Pain?.

[8] Dunlop R., Laming P. (2005). Mechanoreceptive and nociceptive responses in the central nervous system of goldfish (*Carassius auratus*) and trout (*Oncorhynchus mykiss*). J. Pain.

[9] Nordgreen J., Horsberg T.E., Ranheim B., Chen A.C. (2007). Somatosensory evoked potentials in the telencephalon of Atlantic salmon (*Salmo salar*) following galvanic stimulation of the tail. J. Comp. Physiol. A. Neuroethol. Sens. Neural. Behav. Physiol..

[10] Chervova L.S., Lapshin D.N. (2000). Opioid modulation of pain threshold in fish, Doklady. Biol. Sci..

[11] Cunha S.R., Gonçalves R., Silva S.R., Correia A.D. (2008). An automated marine biomonitoring system for assessing water quality in real-time. Ecotoxicology.

[12] Newby N.C., Stevens E.D. (2009). The effects of the acetic acid “pain” test on feeding, swimming, and respiratory of rainbow trout (*Oncorhynchus mykiss*): A critique on Newby and Stevens (2008) - Response. Appl. Anim. Behav. Sci..

[13] Reilly S.C., Quinn J.P., Cossins A.R., Sneddon L.U. (2008). Behavioral analysis of a nociceptive event in fish: comparisons between three species demonstrate specific responses. Appl. Anim. Behav. Sci..

[14] Sneddon L.U., Braithwaite V.A., Gentle M.J. (2003). Novel object test: Examining nociception and fear in the Rainbow trout. J. Pain.

[15] Sneddon L.U., Braithwaite V.A., Gentle M.J. (2003). Do fishes have nociceptors? Evidence for the evolution of a vertebrate sensory system. Proc. R. Soc. Lond. B Biol. Sci..

[16] Chervova L.S., Kamensky A.A., Malyukina G.A., Baturina Y., Deigin E., Yarova E.P. (1992). Investigation of the mechanism of intranasal effect of dermorphin in representative of two classes of vertebrates. Zh. Evol. Biokhim. Fiziol..

[17] Gonzalez-Nunez V., Rodríguez R.E. (2009). The zebrafish: A model to study the endogenous mechanisms of pain. ILAR J..

[18] Sneddon L.U. (2009). Pain Perception in fish: Indicators and endpoints. ILAR J..

[19] Newby N.C., Gamperl A.K., Stevens E.D. (2007). Cardiorespiratory effects and efficacy of morphine sulfate in winter flounder (*Pseudopleuronectes americanus*). Am. J. Vet. Res..

[20] Tukey J.W., McLaughlin D.H. (1963). Less vulnerable confidence and a significance procedures for location based on a single sample: Trimming/Winsorization 1. Sankhya A.

[21] Brunner E., Domhof S., Langer F. (2002). Nonparametric Analysis of Longitudinal Data in Factorial Experiments.

[22] Bowman T., Zon L.I. (2010). Swimming into the future of drug discovery: *in vivo* chemical screens in zebrafish. ACS Chem. Biol..

[23] Zon L.I., Peterson R.T. (2005). *In vivo* drug discovery in the zebrafish. Nat. Rev. Drug Discov..

[24] Le Bars D., Gozariu M., Cadden S.W. (2001). Animal models of nociception. Pharmacol. Rev..

[25] Ashley P.J., Sneddon L.U., McCrohan C.R. (2007). Nociception in fish: stimulus-response properties of receptors on the head of trout *Oncorhynchus mykiss*. Brain Res..

[26] Chervova L.S. (1997). Pain sensitivity and behavior of fishes. J. Ichthyol..

[27] Sneddon L.U. (2003). The evidence for pain in fish: the use of morphine as an analgesic. Appl. Anim. Behav. Sci..

[28] Wheeler-Aceto H., Porreca F., Cowan A. (1990). The rat paw formalin test: comparison of noxious agents. Pain.

[29] Nordgreen J., Garner J.P., Janczac A.M., Ranheim B., Muir W.M., Horsberg T.E. (2009). Thermonociceptions in fish: effects of two different doses of morphine on thermal threshold and post-test behavior in goldfish (*Carassius auratus*). Appl. Anim. Behav. Sci..

[30] Ribeiro M.D., Joel S.P., Zeppetella G. (2004). The bioavailability of morphine applied topically to cutaneous ulcers. J. Pain Symptom Manage.

[31] Ribeiro M., Pinto A., Pinto M., Heras M., Correia A.D., Bardaji E., Tavares I., Castanho M. (2011). Inhibition of nociceptive responses after systemic administration of amidated kyotorphin. Br. J. Pharmacol..

[32] Matthes H.W.D., Maldonado R., Simonin F., Valverde O., Slowe S., Kitchen I., Befort K., Dierich A., Le Meur M., Dolle P., Tzavara E., Hanoune J., Roques B.P., Kieffer B.L. (1996). Loss of morphine-induced analgesia, reward effect and withdrawal symptoms in mice lacking the μ-opioid-receptor gene. Nature.

[33] Stevens C.W. (1996). Relative analgesic potency of mu, delta and kappa opioids after spinal administration in amphibians. J. Pharmacol. Exp. Ther..

[34] Ehrensing R.H., Michell G.F., Kastin A.J. (1982). Similar antagonism of morphine analgesia by MIF-1 and naloxone in *Carassius auratus*. Pharmacol. Biochem. Behav..

[35] Mansour A., Fox C.A., Akil H., Watson S.J. (1995). Opioid-receptor mRNA expression in the rat CNS: anatomical and functional implications. Trends Neurosci..

[36] Barrallo A., Gonzalez-Sarmiento R., Alvar F., Rodriguez R.E. (2000). ZFOR2, a new opioid receptorlike gene from the teleost zebrafish (*Danio rerio*). Brain Res. Mol. Brain Res..

[37] Herrero-Turrion M.J., Rodriguez R.E. (2008). Bioinformatic analysis of the origin, sequence and diversification of mu opioid receptors in vertebrates. Mol. Phylogenet. Evol..

[38] Newby N.C., Wilkie M.P., Stevens E.D. (2009). Morphine uptake, disposition, and analgesic efficacy in the common goldfish (*Carassius auratus*). Can. J. Zool..

[39] Sanchez-Simon F.M., Rodriguez R.E. (2008). Developmental expression and distribution of opioid receptors in zebrafish. Neuroscience.

